# Novel cellular evidence of lipophagy within the Sertoli cells during spermatogenesis in the turtle

**DOI:** 10.18632/aging.101070

**Published:** 2016-10-16

**Authors:** Nisar Ahmed, Yi Liu, Hong Chen, Ping Yang, Yasir Waqas, Tengfei Liu, Jameel Ahmed Gandahi, Yufei Huang, Lingling Wang, Xuejing Song, Imran Rashid Rajput, Taozhi Wang, Qiusheng Chen

**Affiliations:** ^1^ Laboratory of Animal Cell Biology and Embryology, College of Veterinary Medicine, Nanjing Agricultural University, Nanjing, Jiangsu Province 210095, China; ^2^ Faculty of Veterinary and Animal Sciences, LUAWMS, Uthal 90150, Pakistan; ^3^ Faculty of Animal Husbandry and Veterinary Sciences, Sindh Agriculture University, Tando Jam 70060, Pakistan

**Keywords:** spermatogenesis, lipophagy, Sertoli cell, LC3, Chinese soft-shelled turtle

## Abstract

Spermatogenesis is a complex process producing haploid spermatozoa, and the formation of lipid droplets (LDs) within Sertoli cells is critical to maintaining normal spermatogenesis. However, the utilization of LDs within Sertoli cells is still largely unknown. In the present study, proliferation of spermatogonial cells had begun in May, whereas the meiotic cells occurred predominately in July and majority of spermiogenic cells were observed in the seminiferous tubules in October. However, TEM and Oil Red O staining demonstrated that a larger number of LDs had accumulated within the Sertoli cells in May compared to that in October. There were several LDs attached to the isolation membrane/phagophore, suggesting that the LDs may be a source of endogenous energy for the biogenesis of autophagosomes. The LDs were enclosed within the autophagosomes in May, whereas, autophagosomes and mitochondria were directly attached with large LDs within the Sertoli cells in October. Furthermore, immunohistochemistry results demonstrated the stronger localization of LC3 on the Sertoli cells in May than in October. This study is the first to provide clear evidence of the two different modes of lipophagy for lipid consumption within Sertoli cells, which is a key aspect of Sertoli germ cell communication during spermatogenesis.

## INTRODUCTION

Spermatogenesis is a complex process of male gamete production with successive cellular differentiation that occurs with the support of the Sertoli cells. This process consists of the proliferation of progenitor cells (spermatogonial mitosis), which undergo reduction divisions, segregation and recombination of genetic material, known as spermatocytic meiosis, and maturational or spermiogenic phases, which are the final aspect of spermatogenesis [1, 2]. Once the population of germ cells has reached maturation, spermiation consequently occurs, and the germ cells are released as spermatozoa from the seminiferous epithelium within the testes of vertebrates [3-5]. Few studies have described the major steps of spermatogenesis (light and electron microscopy) in more than several species of reptilian taxon, such as the Ophidia (snakes), the temperate lizard (*Podarcis muralis*) and the turtle (*Trachemys scripta*), but even in these studies, the ultrastructure is mainly focused on spermiogenesis and the mature spermatozoon. The ultrastructure of the early stages of spermatogenesis has not been well explored in Reptilia [3, 6, 7], and the ultrastructure of spermatogenesis within the Chinese soft-shelled turtle, *Pelodiscus sinensis*, has not yet been reported, although its spermiogenesis has been observed [8].

Germ cells advance through development in association with Sertoli cells, which play a central role in the control of spermatogenesis. The key functions of Sertoli cells as reported in mammals, are providing structural support, phagocytosing degenerating germ cells, protecting the germ cells, releasing spermatids at spermiation and nourishing the developing germ cells [9, 10]. Sertoli cells are also involved in the formation of LDs, the cytoplasmic stores of neutral lipids, but the existence of these lipid inclusions within Sertoli cells exhibits variation throughout spermatogenesis in rat testes [11]. The utilization of lipids is critical to maintain cellular energy homeostasis for different cellular processes. During nutrient scarcity, cellular lipids stored as triglycerides in LDs are hydrolyzed into fatty acids for energy. Previous studies have found that the formation of LDs is associated with the process of the phagocytosis of residual bodies or apoptotic cells within Sertoli cells, which was detected by Oil Red O staining [12]. Recently, autophagy has also been implicated in neutral lipid utilization from droplets under starvation conditions in mouse hepatocytes [13].

Autophagy is an intracellular degradation system conserved among eukaryotes, and a prominent feature of autophagy is the dynamic membrane reorganization that results in the formation of double-membrane spherical structures known as autophagosomes. The contents of the autophagosome are then degraded by lysosomes, which are primarily needed to supply nutrients. [14]. When this double membrane structure develops around the lipid droplets, it is termed the lipophagosome, and the process is called lipophagy [15, 16]. Autophagy is regularly monitored with the LC3, a specific marker to detect autophagosome by light microscopy. LC3, microtubule-associated protein light chain 3, is a mammalian homolog of yeast Atg8, when autophagosome biogenesis starts, LC3 is conjugated with phosphatidylethanolamine and localizes on the inner and outer autophagic membranes or autophago-somes [17]. Recently, it has become evident that autophagy plays an active role in Sertoli cells [18-21]; it is involved in the clearance of androgen binding protein (ABP) in rat Sertoli cells and in the assembly of ectoplasmic specialization within mouse Sertoli cells [19, 22]. However, the role of autophagy in Sertoli cells is still largely unknown [22].

The objectives of the current study were to establish a detailed analysis of germ cell development strategy and ultrastructure and to explore the in vivo role of lipophagy in the consumption of LDs within Sertoli cells in the testes of the Chinese soft-shelled turtle, *Pelodiscus sinensis*.

## RESULTS

### Light microscopy

**Figure 1:**
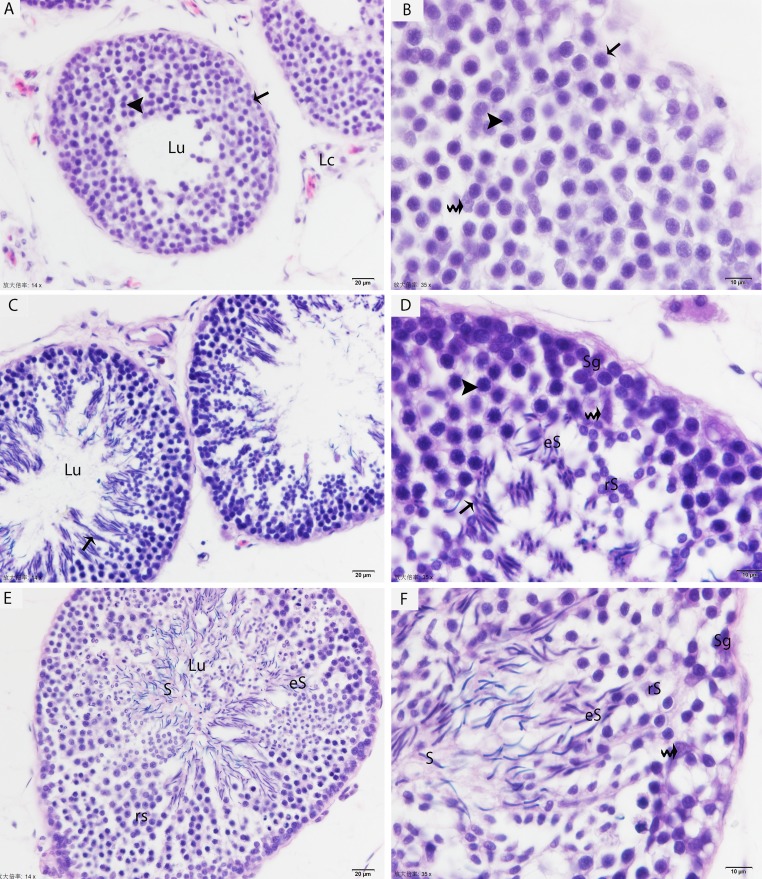
Light micrograph shows the histological structure of testis (**A**, **B**) Seminiferous tubules show the spermatogonia (arrow) and few leptotene spermatocytes (arrowhead) in May. (**C, D**) Pachytene spermatocytes (arrowhead), round/elongated spermatids are present in the seminiferous epithelium, and spermatids are arranged in the sperm column (arrow) in July. (**E**, **F**) The round/elongated spermatids as well as free spermatozoa in the lumen and few spermatogonia are observed in October. LC: Leydig cell; Sg: spermatogonia; rS: round spermatid; eS: elongated spermatid; S: spermatozoa; Lu: lumen; (curved arrow): Sertoli cells. H & E stain. Scale bar= 20μm (**A**, **C**, **E**) and 10μm (**B**, **D**, **F**).

The testis of the Chinese soft-shelled turtle consisted of seminiferous tubules lined with developing germ cells and a permanent number of Sertoli cells. In May, the seminiferous epithelium contained numerous mitotic cells (proliferative spermatogonia) and a few early spermatocytes, which indicated an early stage of spermatogenesis (Figs. 1A, 1B). By July, pachytene spermatocytes were detected in the seminiferous epithelium. The majority of the germ cell population was meiotic cells (spermatocytes) with some round/elongated spermatids progressing through the early events of spermiogenesis. These differentiating sperma-tids were arranged in the sperm column within the pockets of the Sertoli cells, but the lumen of the seminiferous tubule remained clear (Figs. 1C, 1D). In October, the seminiferous epithelium was lined with numerous spermatids that were progressing into various phases of spermiogenesis, and the lumen was filled with free spermatozoa. The meiotic activity of the germina-tive epithelium was largely completed in the seminiferous tubules, and the meiotic cells were drastically reduced at this stage. Few resting sperma-togonia and Sertoli cells were observed around the basal membrane (Figs. 1E, 1F).

### Immunohistochemistry (IHC)

**Figure 2:**
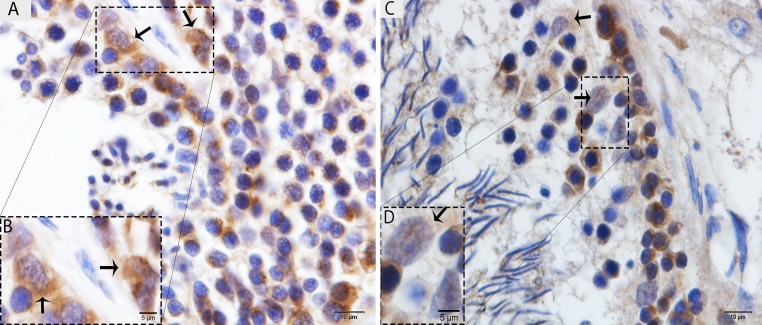
Light micrograph of LC3 localization in the testis (**A**) The immunoreactivity shows strong positive expression on the Sertoli cells in May. (**B**) A higher magnification of the rectangular area. (**C**) Weak, positive expression on the Sertoli cells in October. (**D**) Illustration of rectangular area. Scale bar= 10μm (**A**, **C**) and 5μm (**B**, **D**).

The immunohistochemistry for LC3, which is a specific marker for autophagosomes, was observed in the testis of the Chinese soft-shelled turtle. Immunohistochemist-ry demonstrated strong immunoreactivity in the Sertoli cells in May (Fig. 2A), and weak immunoreactivity was noted in October (Fig. 2B).

### Oil red O (ORO) staining

**Figure 3:**
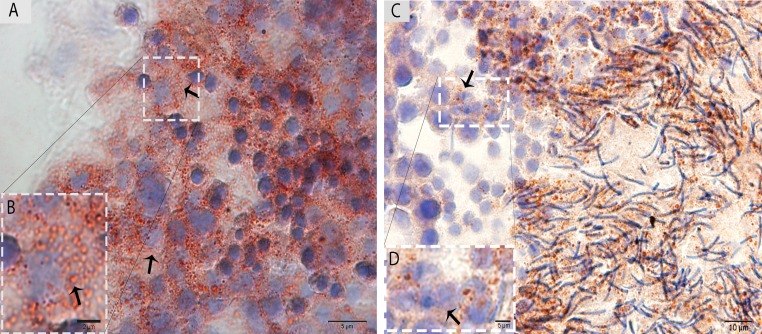
Light micrograph of Oil Red O staining in the testis (**A**)The ORO staining shows a large number of lipid droplets in the Sertoli cells (arrow) in May. (**B**) A higher magnification of the rectangular area. (**C**) Lower numbers of lipid droplets in the Sertoli cells (arrow) in October are observed. (**D**) Illustration of the rectangular area. Scale bar= 10μm (**A**, **C**), 2μm (**B**) and 5μm (**D**).

The ORO staining was performed in the testis for LD analysis in the Sertoli cells during spermatogenesis. The results indicate that a large number of LDs accumulated within the Sertoli cells in May. In contrast, few LDs were observed within the Sertoli cells in October, and more localization was observed around the elongated spermatids at this time (Fig. 3A, 3B).

### Transmission electron microscopy (TEM)

**Figure 4:**
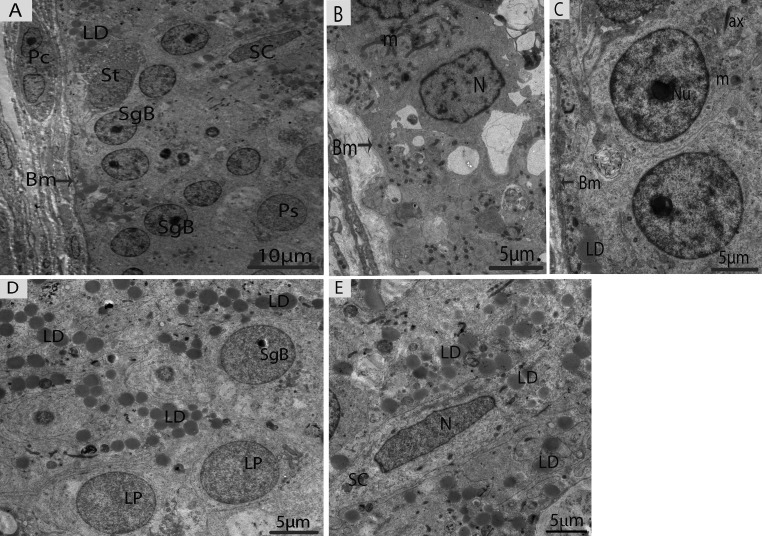
Electron micrograph of seminiferous tubules in May (**A**) Seminiferous tubules contain type B spermatogonia and early spermatocytes. (**B**) Type-A spermatogonia. (**C**) Type-B spermatogonia. (**D**) The numerous lipid droplets are observed around leptotene spermatocytes. (**E**) Sertoli cells contained lipid droplets. Bm: basal membrane; SgB: spermatogonia type B; Ps: primary spermatocyte; SC: Sertoli cell; LD: lipid droplets; Lp: leptotene spermatocyte; N: nucleus; m: mitochondria; ax: axonome; Pc: peritubular cell; St: stem cell. Scale bar= 10μm (**A**) and 5μm (**B**, **C**, **D**, **E**).

**Figure 5:**
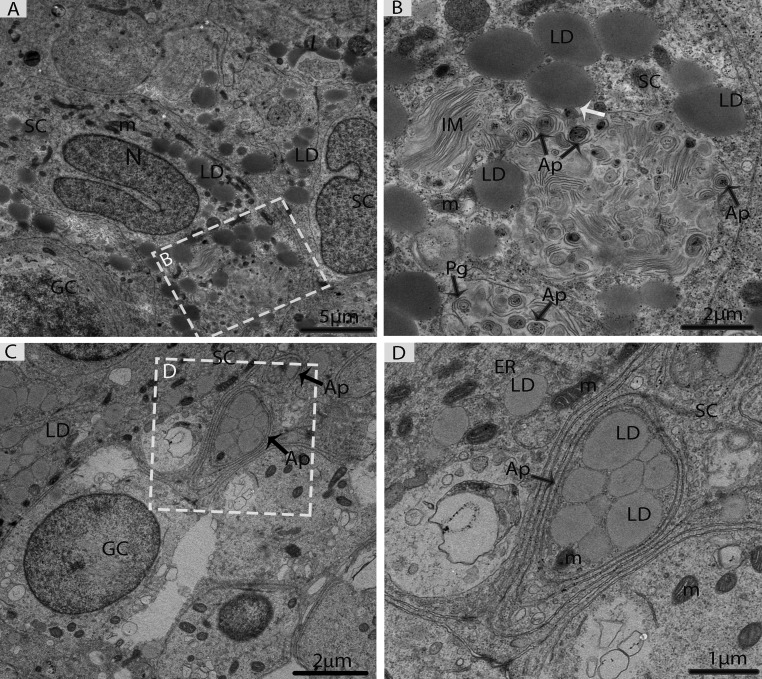
Electron micrograph of Sertoli cells during consumption of lipid droplets via lipophagy in May (**A**) Sertoli cells contained numerous lipid droplets. (**B**) Illustration of Fig. A (rectangular area) shows the lipid droplets in contact with a phagophore and autophagosomes (white arrow). (**C**) Lipid droplets enclosed in the autophagosome. (**D**) Higher magnification of the square from Fig. C. SC: Sertoli cell; LD: lipid droplets; IM: isolation membranes; Ap: autophagosome; Pg: phagophore; GC: germ cell; m: mitochondria; ER: endoplasmic reticulum. Scale bar= 5μm (**A**), 2μm (**B**, **C**) and1μm (**D**).

In May, three different types of spermatogonia were observed in the seminiferous epithelium, such as stem cells, type-A spermatogonia and type-B spermatogonia. The large population of these spermatogonia cells was accumulated near the basement membrane in the seminiferous epithelium. The stem cells appeared to be the smallest of the spermatogonia with their nuclei ovoid in shape without a nucleolus. The type-A spermatogonia were observed as ovoid in shape along with nuclei, which contained very little heterochromatin and had one flattened cellular surface resting directly on the basement membrane. The type-B spermatogonia contained round nuclei, including a prominent nucleolus and several large globules of heterochromatin within the nucleoplasm (Figs. 4A, 4B, 4C). A few early spermatocytes were also observed in the May samples, along with a large number of LDs. These LDs originated from the Sertoli cells during this phase of spermatogenesis (Figs. 4D, 4E, 5A). Several large LDs were detected around the isolation membranes/phagophore within the Sertoli cells during the development of autophagosomes (Figs. 5A, 5B). Then, the autophagosomes enclosed several LDs and mitochondria (Figs. 5C, 5D).

**Figure 6:**
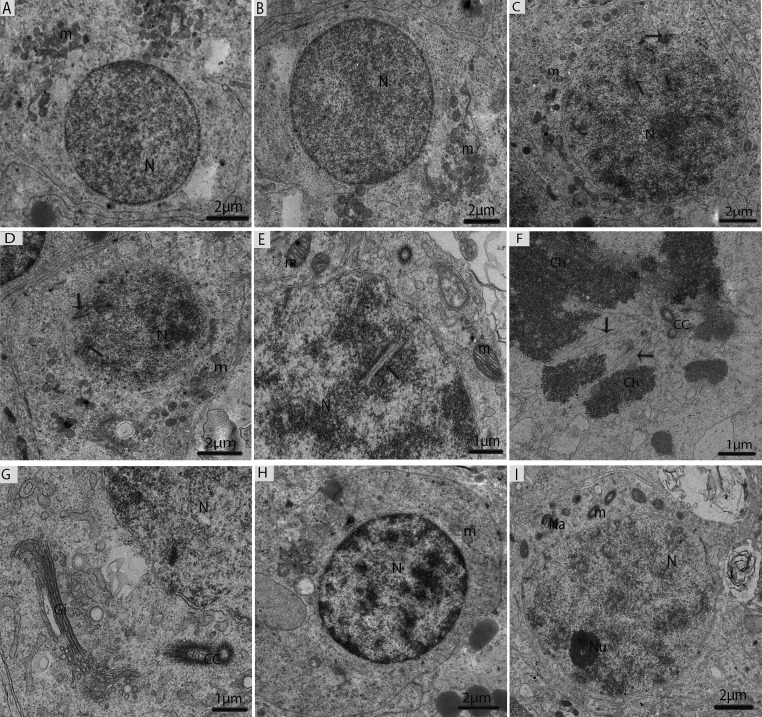
Electron micrograph of meiotic cells in July (**A**) Primary spermatocytes in pre-leptotene stage. (**B**) Primary spermatocytes in leptotene. (**C**) Primary spermatocyte in zygotene beginning to form synaptonemal complexes (arrow). (**D**) Primary spermatocyte with visible synaptonemal complexes (arrow) in pachytene. (**E**) Synaptonemal complexes (arrow). (**F**) Thicker and dense chromosomes are attached with spindle fibers (arrow). (**G**) Golgi complex is observed close to the centrosome. (H) Primary spermatocytes in diplotene. (**I**) Secondary spermatocytes containing vacuolated mitochondria, including a few nuages. N: nucleus; m: mitochondria; Ch; chromosomes; CC: centrosome; Na: nuage; Gi: Golgi apparatus. Scale bar= 2μm (**A**, **B**, **C**, **D**, **H**, **I**) and 1μm (**E**, **F**, **G**).

Type-B spermatogonia undergo mitotic divisions to produce primary spermatocytes (pre-leptotene), which are characterized by increasing nuclear and cytoplasmic size and the gradual condensation of nuclear chromatin into distinct chromosomes. Pre-leptotene spermatocytes contained a round nucleus and numerous mitochondria accumulated in one place (Fig. 6A). In contrast, leptotene spermatocytes appeared slightly larger than the pre-leptotene spermatocytes, and their nuclei contain fine filamentous chromatin fibers (Fig. 6B). The zygotene spermatocytes were noted by an increase in the thickening of the filamentous chromatin fibers within the nucleus and the synaptonemal complexes that started to appear (Fig. 6C). Pachytene spermatocytes contained well-developed synaptonemal complexes, which connected the homologous chromosomes during the first meiotic divisions. Thicker and dense chromosomes were attached with spindle fibers along with the centriole and the Golgi complexes that were found close to the centrosome (Figs. 6D, 6E, 6F, 6G). The diplotene spermatocytes appeared with thick fully condensed chromosomal fibers that were interspersed with large open areas of nucleoplasm (Fig. 6H). Meiosis I progressed to produce secondary spermatocytes, which appeared with nuclei along with nucleolus. There were few nuages that developed, and vacuolated mito-chondria were observed in the secondary spermatocytes (Fig. 6I).

**Figure 7:**
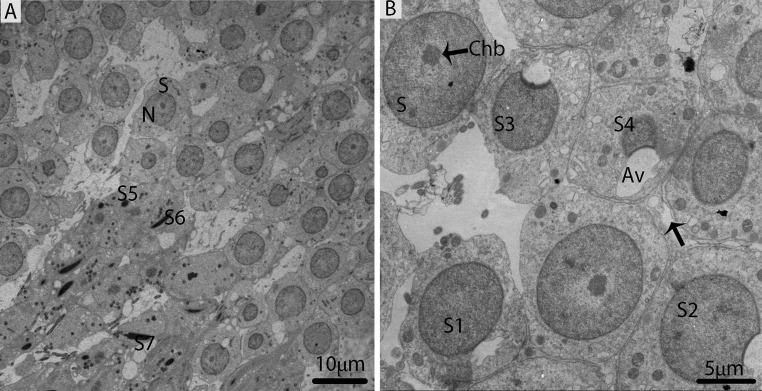
Electron micrograph of seminiferous tubules in October (**A**) The majority of cell types within the seminiferous tubules are round/elongated spermatids. (**B**) Different stages of spermiogenic cells. S: spermatid; eS: elongated spermatid; SC: Sertoli cell; Sp: spermatid; Av: acrosomal vesicle; Chb: chromatin body; (arrow). Scale bar= 10μm (**A**) and 5μm (**B**).

In October, the majority of germ cell types within the seminiferous epithelium were spermiogenic cells (round or elongated spermatids), which were characterized by the development of the acrosomic system, the elongation of the nucleus, and the condensation of chromatin material, as suggested by Russell [4]. The spermatids appeared with spherical nuclei, which were centrally located and contained chromatin bodies (Fig. 7A). The 7steps of spermatids were observed during spermiogenesis; the first four steps of round spermatids (S1, S2, S3, and S4) represent the acrosome phase of development. The acrosomal vesicle was in direct contact with the nuclear envelope and pressed against the nuclear wall, in which it formed a wide shallow depression. This depression progressively increased in the nucleus from Step1 to Step2, Step3 and Step4 spermatids. Meanwhile, the elongation and condensation of chromatin within the nucleus characterized Step5, Step6 and Step7 spermatids. These spermatids existed in the bundles within the pockets of the Sertoli cells, consequently forming the sperm column (Figs. 7A, 7B). During the spermiogenic phase, the processes of the Sertoli cells contained several lipid droplets, autophagosomes and abundant mitochondria.

**Figure 8:**
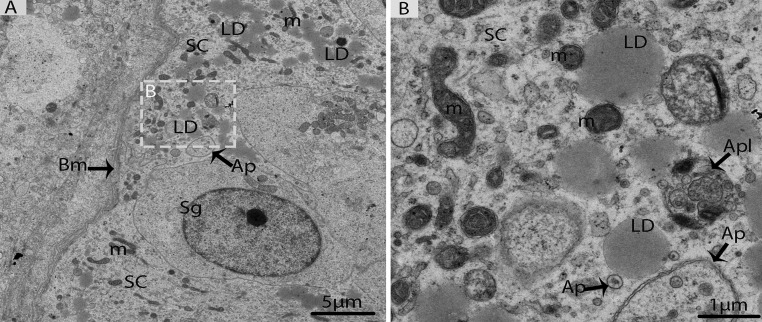
Electron micrograph of Sertoli cells in October (**A**) Sertoli cells appeared with lipid droplets mitochondria and autophagosomes. (**B**) Illustration of panel **A** (rectangular area) clearly shows the mitochondria and autophagosomes attached to lipid droplets. SC: Sertoli cell; Sg: spermatogonia; Bm: basal membrane; Ap: autophagosome; Apl: autophagolysosome; LD: Lipid droplets; m: mitochondria. Scale bar= 5μm (**A**) and1μm (**B**).

These autophagosomes and mitochondria were attached to the large LDs (Figs. 8A, 8B). Detailed descriptions about spermiogenesis have been described in an earlier study by our research group [8].

## DISCUSSION

Spermatogenesis within the seminiferous epithelium of the Chinese soft-shelled turtle, *Pelodiscus sinensis*, was closely parallel to that previously reported in the slider turtle [23]. In the present study, the proliferation of germ cells started in May; meiotic cell division peaked in July, and spermiogenesis largely was complete in October. Consequently, spermiation occurred in November with a remaining period of spermatogenic quiescence (December to April) [24]. The sperm development in the *Pelodiscus sinensis* seemed to be a postnuptial pattern, which is consistent with the pattern observed in *A. ferox* and *Trachemys scripta* [25]. In contrast, although spermatogenesis within most lizards follows a prenuptial pattern of development, it is followed by a comparatively shorter quiescence period [26]. Germ cell development starts in midsummer, continues throughout winter and is completed by the following June in the lizards [6, 27]. This divergence in timing probably resulted from different environmental conditions related with temperature in the respective regions of the animals studied.

The majority of the soft-shelled turtle's germ cell population progressed through the phases of spermatogenesis (proliferation, meiosis, and spermiogenesis) as a single cohort within the seminiferous epithelium. Furthermore, the germ cell development strategy of this turtle was found to be temporal in nature, which is consistent with that found in other temperate-zone reptiles, (wall lizard, black swamp snake, slider turtle, and alligator) [6, 7, 23]. In contrast, all other amniotic germ cell development strategies (birds and mammals) result in waves of sperm being released throughout the active months of the testes [4, 28, 29]. In the current study, five to six generations of spermatids within the seminiferous epithelium were observed at the same time in October, which is much different than that found in birds and mammals [4]. These elongating steps of spermiogenesis were apically accumulated within the pockets of the Sertoli cell processes and were released in the lumen with spermiation [24]. Moreover, we observed numerous LDs within the Sertoli cells in May than in October by TEM and ORO staining, and our previous study reported that the Sertoli cells contain abundant LDs during early spermatogenesis as compared to late spermatogenesis [30]. These LDs can be used for nourishing the germ cells. However, it was found that the Sertoli cell lipids were absorbed into spermatids during the different stages of spermatogenesis and that lipid inclusions can be transferred from the Sertoli cells to primary spermatocytes [12, 16, 31]. Many reptilian Sertoli cells undergo a well-defined lipid cycle that corresponds to spermatogenic activity, but the metabolism of lipids in the Sertoli cells remains an open question [12, 32, 33].

We found, for the first time, the close contact of numerous LDs with the isolation membranes/the phagophore during the formation of autophagosomes within Sertoli cells (Fig. 4B). When two organelles come into contact in kiss-and-run events, it may allow a direct exchange of the material [34]. Hence, we suggested that the LDs regulate autophagosome biogenesis by donating lipids to the outside membrane of the phagophore, as suggested by Dupon and his coworkers [34]. The mechanism of autophagosome biogenesis is a remarkable cell biology problem because it stands apart from the canonical vesicular trafficking process [35-37]. Shpilka et al. [37] further reported that the order in the relationship between autophagy and LDs, instead of being a substrate for lipophagy, is that the LDs act as contributors for autophagosome biogenesis in kiss-and-run events.

Another widely accepted relationship is that autophagosomes can engulf LDs in a process known as “lipophagy” [16], which includes lysosomal lipolysis and the release of fatty acids for metabolic needs [38, 39]. Current findings provided clear evidence that several LDs were enclosed within the double membrane autophagosomes in the Sertoli cells in May (early spermatogenesis) (Fig. 4C). In other words, auto-phagosomes and mitochondria were directly attached to the LDs in October, which suggests that lipophagy is involved in lipid metabolism to release endogenous energy for the developing germ cells in two different ways because lipid droplets always provide energy to different cells [16, 40]. Furthermore, our immuno-histochemistry findings demonstrate the stronger positive expression of LC3 in the Sertoli cells in May than in October, which indicates that autophagy is persistent throughout the process of spermatogenesis, but the number of autophagosomes within Sertoli cells is reduced in late spermatogenesis (October). Moreover, Akiko et al. [41] suggested that electron microscopy, along with LC3 localization, can confirm the presence of autophagosomes. Hence, our LC3 localization and TEM findings are inline regarding the existence of autophagosomes within the Sertoli cells.

In conclusion, the mode of spermatogenesis in the Chinese soft-shelled turtle, *Pelodiscus sinensis,* is temporal in nature and is also commonly found in other temperate-breeding reptiles. Moreover, this study provides novel morphological evidence on the involvement of autophagy in the mobilization of LDs within Sertoli cells in two different ways. Several LDs were found during autophagosome biogenesis in the Sertoli cells. The autophagosome enveloped several LDs in May (early spermatogenesis), while it was directly attached to LDs in October (late spermatogenesis), implying that this process becomes a source of endogenous energy for the different spermatogenic events. This research describes a new role of lipid metabolism through lipophagy within Sertoli cells during the process of spermatogenesis, and understanding this particular set of relationships may be highly valuable.

## MATERIALS AND METHODS

### Animals

15 mature male (3-4 years-old) Chinese soft-shelled turtles *Pelodiscus sinensis* were captured from an aqua farm in Nanjing, Jiangsu province of China in May, July and October, five turtles during each time period. The animals were rendered comatose using intraperitoneally administered sodium pentobarbital (20 mg/animal) and were sacrificed by cervical dislocation. The testes were collected immediately and fixed to performed different techniques (details below). Sample preparation was conducted according to accepted international standards and was approved by the Ethics Committee for Animal Care and Use by the Science and Technology Agency of Jiangsu Province (SYXK (SU) 2010-0009).

### Light microscopy

The testis samples were placed in 10% neutral buffered formalin for fixation overnight, and then embedded in paraffin wax. Sectioning was done at 5μm. These sections were stained with hematoxyline and eosin procedures (Harry's hematoxyline for 2 min and 1% eosin for 30 sec) for light microscopic analysis using an Olympus microscope (BX53), camera (Olympus DP73), Japan.

### Immunohistochemistry (IHC)

The immunohistochemical staining for LC3 was performed according to the manufacturer's recommendations and as suggested in previous studies [42, 43]. Testis specimen sections were processed using a standard immunohistochemistry protocol, as previously described [44]. Briefly, after deparaffiniza-tion, blocking endogenous peroxidase, microwave antigen retrieval, and BSA (Bull Serum Albumin) blocking, the primary antibody of rabbit polyclonal anti-LC3B (ab48394, Abcam, Cambridge, UK) was applied for 1 h at room temperature. After washing with PBS, the slides were incubated for 30 min with goat anti-rabbit-biotinylated antibody. The peroxidase was visualized with DAB, and sections were counter stained with hematoxyline. Sections incubated in PBS alone served as negative controls.

### Oil Red O (ORO) staining

For ORO staining, samples of the testis (May and Oct.) were washed 255 with PBS, fixed with 4% formaldehyde for 10 min, and stained with ORO (Sigma) 256 solution (oil O-saturated solution in isopropanol: water, 3:2) for 15 min as previous 257 description. Then, the sections were washed with 70% alcohol for 5s to remove 258 background staining.

### Transmission electron microscopy (TEM)

The specimen of testis were cut into small parts (1 mm^3^), and fixed into 2.5% glutaraldehyde in PBS (4°C, pH 7.4, 0.1 M) for 24h. Specimen were rinsed in the same PBS and then post-fixed for 1h. at room temperature in the same way by using buffered 1% osmium tetroxide (Polysciences Inc. Warrington, PA, USA) and then washed in the buffer. The samples were dehydrated in ascending concentrations of ethyl alcohol, infiltrated with a propylene oxide–Araldite mixture and then embedded in Araldite. The blocks were then sectioned by using an ultramicrotome (Reichert Jung, Wien, Austria) and the ultrathin sections (50 nm) were mounted on copper coated grids. The specimens were stained with 1% uranyl acetate and Reynold's lead citrate for 20 min. Finally specimen were examined and photographed by using a high resolution digital camera (16 mega pixel) connected to the TEM (Hitachi H-7650, Japan).
